# Nutrient and Mineral Profile of Chosen Fresh and Smoked Fish

**DOI:** 10.3390/nu11071448

**Published:** 2019-06-26

**Authors:** Bożena Kiczorowska, Wioletta Samolińska, Eugeniusz R. Grela, Marta Bik-Małodzińska

**Affiliations:** 1 Institute of Animal Nutrition and Bromatology, University of Life Sciences, Akademicka Street 13, 20-950 Lublin, Poland; 2 Institute of Soil Science, Environment Engineering and Management, University of Life Sciences in Lublin, 20-950 Lublin, Poland

**Keywords:** freshwater fish, sea fish, smoking process, nutrients, minerals, dietary intake

## Abstract

In the present study, were determined the basic nutrients (dry matter, crude ash, crude protein, ether extract, and energy) and mineral elements content in chosen species of raw and smoked freshwater and sea fish. The content of dry matter, and basic nutrients and Na+, K+, Ca^+2^, Mg^+2^, P^+2^, Zn^+2^, and Cu^+2^ in the fish samples was determined. The dietary intake of several macro- and microconstituents per one serving (150 g fresh or smoked fish) was calculated. The fresh fish contained on average 220.2 to 283.7 g·kg^−1^ of dry matter, 12.4 to 10.7 g·kg^−1^ of crude ash, 176.2 to 173.5 g·kg^−1^ of crude protein, 32.6 to 78.6 g·kg^−1^ of ether extract, and 104.6 to 119.1 kcal (freshwater and sea fish, respectively). Thermal treatment reduces the water and fat content in fish meat. Reduction of the K, Ca, Mg, P, Zn, and Cu levels was observed most frequently. The one serving of fish covers approximately 23% and 12% of the recommended dietary amount of K, 7.5–5.0% of Ca, ~12% of Mg, 6.8 to 12.5% of Zn, and about covered 6.7% of Cu. The smoking process increased the concentration of some basic nutrients and reduced the fat and mineral content. Whitefish, trout, halibut, mackerel, and herring had the highest levels of the analyzed minerals.

## 1. Introduction

Over 80% of world fish production is used for consumption. The demand for fish is estimated to grow on all continents and reach even 17.9 kg per capita by 2020 [1]. The increasing consumer awareness of nutritional issues and appropriate composition of meals contributes to the growing interest in health-enhancing food. Fish are perceived as a significant component of a balanced and healthy diet, primarily due to the low fat content compared to animal meat and the content of fatty acids exerting a positive effect on the human organism, e.g., eicosapentaenoic acid (EPA) and docosahexaenoic acid (DHA) [2,3]. Freshwater fish are especially valuable in this respect. Investigations of 27 species of wild-living fish have confirmed the high content of branched chain fatty acids (BCFA) in their meat [4]. The mean BCFA content across all species was on average 1.0% of total fatty acids in edible muscle. The EPA and DHA content constituted 28% ± 7% of total fatty acids. Fish skin had significantly higher BCFA content than muscle tissues, i.e., 1.8% ± 0.7%, but lower EPA and DHA content. Fish are also a good source of protein. The awareness of consumers in this area is low. To ensure protein in the diet, consumers usually choose animal meat [5] or legume seeds [6]. The content of protein and other nutrients in fish meat may vary between species, sex, and even the season of the year [7]. Emre et al. [7] reported that the total protein content in fish ranged from 63.80% to 78.15% and the total fat content varied from 4.57% to 21.29% in different seasons. Fish are also a good source of bionutrients, e.g., vitamins and minerals in diets, although consumers are more likely to choose fruit [8], herbs, and spices [9], or even herbal and slimming teas [10] as food products that are rich in these nutrients. Mohanty et al. [11] investigated the micronutrient composition of 35 food fish from varying aquatic habitats. The analysis showed that sea and ocean fish were rich in sodium and potassium and small, indigenous fish were rich in calcium, iron, and manganese. High selenium and phosphorous contents were determined in coldwater fish and in brackish water fish, respectively.

Investigations of the impact of nutrients on consumers’ health are often conducted on raw food, disregarding culinary processes applied to the food products. Thermal treatment of food before consumption is important and sometimes even necessary for healthy nutrition, as it increases consumption safety and food digestibility. Food processing methods, in particular high temperature, have a substantial effect on the maintenance or modification of food structure as well as loss of nutrients. Heat effects resulting in reduced fish water activity allow better preservation causing microbial sterilization and thus minimize spoilage and increase the shelf life of fish products. They contribute to changes in the chemical composition and, hence, the nutritional value of processed food [12].

Many authors have studied the influence of various methods of culinary processing, mainly boiling and baking, on the nutrient composition most frequently performing analyses of the content and quality of fish fat or protein. During the smoking process, fats and water drip from the fish, resulting in the physical loss of lipids, protein, and micronutrients. Smoking at high temperatures can also reduce the functionality of essential amino acids. Smoke particles can react with nutrients in fish meat and may lead to loss of important nutrients and antioxidants [13]. Literature reports are typically focused on several most popular species of fish, e.g., salmon, mackerel, sardine, anchovy, tilapia, etc. [12,13]; Cieślik et al. [14] observed that the process of smoking of freshwater fish: common carp, rainbow trout, and northern pike led to an increase in almost all amino acids, with the highest amount of EAA. However, Famurewa et al. [15] observed increasing content of protein at a level of 5.5% and crude ash—~14% as well as decreasing fat content—as high as 27% during fish smoking. In processed tilapia, significant changes in the ash content from 11.12% (fresh) to 14.72% (traditionally smoked) were observed as well. The mineral content did not show any significant differences (*p* > 0.05) [16]. However, it is difficult to find a comprehensive study providing a comparison of the chemical composition and nutritional value of many freshwater and sea fish species as well as the content of micronutrients and analysis of the impact of culinary methods on changes in nutrient compounds.

Therefore, the present study attempted to determine the content of basic nutrients and mineral elements in some species of raw and smoked freshwater and sea fish.

## 2. Materials and Methods

### 2.1. Fish Material and Smoking Conditions

The investigations were conducted on 6 freshwater fish species and 7 sea and ocean fish species. Whole fresh fish were purchased in a specialist shop in 2016 (Lublin, Poland). As specified by the seller, the freshwater fish originated from a fish farm localized in the south and east of Poland, and the sea fish were caught in the Baltic Sea, the Mediterranean Sea, and the Atlantic Ocean. Salmon was obtained from an aquaculture farm, where water recirculation systems were used to meet the fish farming requirements. Therefore, the salmon was assigned to the group of freshwater species originating from fish farms. The characteristics of the analyzed fish and the experimental design are shown in Table 1.

Prior to the smoking treatment, the fresh fish were kept in a 6% salt bath for approximately 16 h. The fish were smoked in an electric smoking chamber in a fish processing plant (KwG-3-E-D1-S1, Stawiany, Pruszcz Gdański) in accordance with the standards of the EU Commission Regulations No. 835/2011 [17]. The process of hot smoking consisted of two stages: drying at a temperature of 60 ± 2 °C for 140 min and smoking at 60 ± 2 °C for 90 min, with temperatures of 46 °C and 51 °C in the geometric center of the fish carcass. After cooling, each fish was vacuum-packed separately in a PA-PE plastic bag and transferred into the laboratory at a temperature of +4 ± 1 °C.

### 2.2. Basic Composition and pH Measurement

The chemical analyses involved 10 fresh fish of each species and 10 smoked fish of each species. Within one species, the fish were selected based on uniform size and weight. The differences in the weight and length between individual fish did not exceed 5% of the mean value determined in 30 fish of a species available at the place of purchase. Fresh and smoked carcasses were filleted and skinned. Two samples were collected from each individual and subjected to chemical analyses performed in triplicate after averaging (the meat was minced with a hand blender). The content of dry matter (Method 44-15A) and basic nutrients (crude ash—Method 08-01, crude protein—Method 46-06, ether extract (crude fat determined with the Soxhlet method)—Method 30-10) in the fish samples (250 g of each fish) was determined according to standard AOAC [18] procedures.

The energy value of the analyzed fish meat is based on the Atwater general factors for the energy density of fat and protein (9 and 4 kcal·g^−1^, respectively) [19]. The energy value of the fish meat expressed in kcal was converted to kJ with a coefficient of 4.1868.

The pH value was determined by dipping a pH electrode into homogenates of filleted fish in distilled water (1/1) [20]. All measurements were performed at room temperature using a pH-meter (WTW Inolab, Weilhem, Germany).

### 2.3. Determination of Mineral Elements

The chemical analysis involved determination of the content of Na+, K+, Ca^+2^, Mg^+2^, P^+2^, Zn^+2^, and Cu^+2^ in mineralized fish meat samples (*n* = 3). The contents of the elements were determined in the fish materials (3 g of fish meat) after incineration in a muffle furnace at 480 °C. The resultant ash was solubilized on crucibles using 6 mol l^−1^ of spectrally pure hydrochloric acid (POCH, Poland). Na and K were analyzed using flame atomic emission spectroscopy (FAES) with a flame photometer (Pye Unicam SP 2900, Cambridge, UK) at a wavelength of λ = 589.0 nm and λ = 766.5 nm, respectively. Ca, Mg, Zn, and Cu were determined using flame atomic absorption spectroscopy (FAAS) with a SOLAAR 939/959 spectrophotometer (Unicam, Cambridge, UK). Calcium was determined at λ = 422.7 nm, magnesium at λ = 285.2 nm, zinc at λ = 213.9 nm, and copper at λ = 324.8 nm, according to the Polish Norm PN-EN ISO 6869:2002 [21]. The accuracy of the analytical procedure was verified by an analysis of certified reference materials for Multielement Trace Analysis Fish Tissue (IAEA-407, Atomic Energy Agency, Vienna). The recovery levels (*n* = 3) and relative standard deviations (RSD) for the analyzed elements were Na (100.2%, 4.1%), K (99.2%, 5.3%), Ca (97.5%, 6.8%), Mg (98.4%, 4.9%), Zn (97.3%, 6.8%), and Cu (97.5%, 6.1%). The phosphorus content was determined with the spectrometric method at 400 nm using a Helios Alpha UV-VIS apparatus (Spectronic Unicam, Leeds, UK), according to AOAC [19].

### 2.4. Nutritional Content of Fish Per Serving

The intake of proximal nutrients: protein, fat, energy, and some minerals: Na, K, Ca, Mg, P, Zn and Cu per one serving of the fresh and smoked fish were calculated. One serving of fish was assumed to weigh 150 g, based on the average weight of a filleted fish of various species recommended for consumption by the Department of Health and Human Services and U.S. Department of Agriculture [3].

### 2.5. Statistical Analysis

The analyses were performed in triplicate and all data were expressed as means. All the data were analyzed with the Statistica software version 10.0. The normality of data and homogeneity of variances were tested using the Shapiro–Wilk and Brown–Forsythe tests, respectively. All data were subjected to multivariate analysis of variance (MANOVA) using general linear model (GLM) procedures with two fixed factors (smoking and the environment of fish occurrence) as well as their interaction. For MANOVA analysis, the Wilk test was used for evaluation of significant effects. One way analysis of variance (ANOVA) or nonparametric Kruskal–Wallis test (a nonparametric equivalent of one-way analysis of variance) was used to determine significant differences between both groups and individual components at a confidence level of *p* < 0.05.

## 3. Results and Discussion

### 3.1. Basic Nutrients, Energy, and pH of Fish Meat

The basic chemical composition and energy value of the fresh and smoked freshwater and sea fish are presented in Table 2. In global effects (MANOVA), the fixed effects and interaction showed differences (*p* < 0.05) for all performance variables. The fresh fish contained on average 220.2 to 283.7 g·kg^−1^ of dry matter, 12.4 to 10.7 g·kg^−1^ of crude ash, 176.2 to 173.5 g·kg^−1^ of crude protein, 32.6 to 78.6 g·kg^−1^ of ether extract, and 104.6 to 119.1 kcal (freshwater and sea fish, respectively). The pH values in fresh fish meat were similar for the freshwater sea fish (6.2 and 6.4, respectively). The highest level of crude ash was detected in trout (FTF), whereas the highest protein content was determined in bream (FBF) and sea bream (SBF). Salmon (FSAF) and halibut (SHAF) exhibited the greatest amount of fat. In turn, crucian (FCF) and flounder (SFF) were characterized by the lowest energy values (70.5 and 68.2 kcal, respectively). Gokoglu et al. [22] determined similar fat content in fresh trout meat, i.e., ~3.88% ± 0.73. However, Erkan and Özden [23] reported moisture content in the raw material of sea bream at the level of 63.52 ± 0.18%, lipid −15.11 ± 0.08%, and crude ash −1.14 ± 0.10%.

The smoking process increased the concentration of nutrients, including the content of dry matter, crude ash, and crude protein, and reduced the fat content; however, it did not always decrease the energy value of the processed fish (Table 2).

The smoked fish were characterized by average 268.1–330.8 g·kg^−1^ of dry matter, 27.6–18.3 g·kg^−1^ of crude ash, 216.9–220.1 g·kg^−1^ of crude protein, 38.0–69.7 g·kg^−1^ of ether extract, and 116.1–150.8 kcal (freshwater and sea fish, respectively). Thermal treatment reduces the water and fat content in fish meat. The present study shows an effect of drying the fish meat, i.e., reduction below 30%, during the smoking process, which was probably associated with the relatively low temperature applied in the process, i.e., 60 °C. Bainy et al. [1] observed an approximately 10% decrease in the moisture content of fish burgers grilled at a temperature of 76 °C. In turn, Bastías et al. [24] reported a decline in the moisture content of approximately 65% in salmon and 75% in mackerel in their investigations of the effect of steam-reinforced thermal culinary processing. Simultaneously, they noted increased protein content and significantly reduced fat content. In turn, the same level of crude ash was noted regardless the type of processing. The nutritional value of the product is determined not only by the parameters and type of culinary treatment but also by the type of processed fish. The boiling, baking, or grilling treatments applied to striped snakehead fish by Marimuthu et al. [25] did not induce significant changes in the fat content. They found that the thermal factor caused a significant increase only in the protein and crude ash content in the grilled fish fillets. This result is consistent with that reported by Bochi et al. [26], who also observed an increase in protein content after cooking. Similar results were found by García-Arias et al. [12], who observed an increase in the protein content in sardine fillets after baking in a conventional oven. In addition, the authors found that the lipid content of the fillets decreased after baking.

In the present study, there were no significant changes in the pH value in the smoked fish in comparison with the fresh fish. Similarly, Bainy et al. [1] and Vanitha et al. [27] did not find a significant impact of different methods of processing of fish and their products on this parameter. This is confirmed by investigations reported by other authors as well. The pH value in raw fish was slightly lower than that in processed fish, but it was still in the range from 6.1 to 6.5 pH in tilapia fillets, from 6.1 to 6.8 pH in Catla fish burgers, and from 5.6 to pH 7.0 in rainbow trout fish hamburgers stored in refrigeration conditions for 21 days [27].

### 3.2. Mineral Elements

The analyzed fish meat differed in the content of the assessed macro- and microelements, depending on the species (Table 3). The MANOVA results allowed concluding that the processing system and the environment of fish occurrence and their interactions had a significant effect on all variables analyzed (*p* = 0.001, *p* = 0.016, and *p* = 0.08 obtained with the Wilk’s test, respectively). The differences observed in the concentration of various nutrients in the analyzed fish species result from not only species variability in the accumulation of mineral elements in tissues but also nutrient availability in the aquatic environment or feed as well as the ability to absorb and transform the compounds into essential nutritional components [28].

The level of Na in the freshwater fish ranged from 0.27 to 0.52 g·kg^−1^ (FSIF to FSAS) (Table 3). It was nearly two-fold higher in the sea fish and ranged from 0.53 to 1.12 g·kg^−1^ (SFF to SMF, respectively). Similar Na levels in fresh fish tissues were reported by Gokoglu et al. [22] and Erkan and Özden [23]. All smoked fish were characterized by several-fold higher Na amounts than in the fresh fish. The Na content increased 7–12-fold in the freshwater fish and 2–6-fold in the sea species, compared with the unprocessed fish. This was associated with the salting treatment applied before the smoking process. The low-fat fish, mostly the freshwater species, accumulated more Na in tissues (FSIS, FTS, and FWS), whereas the high-fat fish were found to accumulate several-fold lower amounts of this element (SHAS, SHES, and SMS). Potassium and phosphorus were the dominant macroelements determined in the fish meat. The K content ranged from 1.4 to 5.09 g·kg^−1^ (SFF to FWF) in the fresh fish and the level of *p* was in the range of 1.18 to 2.87 g·kg^−1^ (SFF to FWF).

The analyzed fish exhibited varied amounts of Ca and Mg in the range of 0.15 (SMF) to 1.03 (FCF) and 0.16 (SFF) to 1.09 (FSAF) g·kg^−1^. Zn was the most abundant microelement in the fish meat and its level substantially varied between the fish species. Its content ranged from 3.83 (SRF) to 9.95 (FWF) mg·kg^−1^. The Cu levels determined in the analyzed fish were considerably lower, i.e., in the range from 0.11 (SCF) to 0.92 (SHEF) mg·kg^−1^. These results confirm the very high biological variability in the levels of mineral elements in freshwater and sea fish. The results are in agreement with reports presented by other researchers [22,23,25].

The smoking process contributed to multidirectional changes in the content of mineral elements in the fish. Reduction of the K, Ca, Mg, P, Zn, and Cu levels was observed most frequently. Compared to the fresh fish, the largest (*p* < 0.05) losses of K were noted in the case of sea bream (by 33%, SBF). In turn, Ca was reduced most substantially in bream and sea bream (on average by 63%, FBS and SBS), Mg and P in trout (by 46% and 59%, respectively, STS), Zn in crucian and herring (on average by 30%, FCS and SHES) and Cu in trout, cod, and mackerel (on average by 17%, FTS, SCS, and SMS). In some fish, there was also a controversial increase in the level of some mineral elements in single variants, e.g., K in herring and mackerel (on average by 31%, SHES, SMS), Ca in herring (by 10%, SHES), and Mg in cod (by 26% SCS). There was no smoking-induced significant increase only in the levels of P, Zn, and Cu in the fish meat.

Literature provides reports of various changes in the content of mineral elements in fish induced by culinary treatment. Thermal processing of fish without an additional supply of mineral elements, e.g., with salt, can reduce the concentrations of Na, Ca, Mg, Zn, and Cu [29]. Similarly, Ersoy and Ozeren [30] found lower P content in fried, baked, and boiled fish fillets than in fresh fish meat. In turn, Marimuthu et al. [25] indicated that mineral contents in snakehead fish increased depending on the cooking methods. They found higher Ca, Zn, and Cu concentrations in samples cooked at 160 °C than those cooked at 180 °C. The Zn levels determined in boiled, baked, fried, and grilled trout fillets were higher than those in raw fish meat [22]. Previous studies demonstrated that processing and cooking methods had little or no effect on the mineral composition of fish. Such information can also be found in current studies. The absence of an effect of the fish processing methods has most frequently been reported for the levels of K, Ca, and Mg [25], Fe [30], and Cu [13,25,30].

### 3.3. Calculated Dietary Intake by Consuming One Serving of Fresh and Smoked Fish

The percent coverage of daily supply for selected nutrients and macro and microelements by consuming one serving (150 g) of fresh and smoked fish was calculated and listed in Table 4. The results demonstrate that the consumption of one serving of fresh fish provides basic nutrients in the range of approximately 12–21.7% of daily protein (FCF - SBF) and 0.79–19.85% of daily fat (FTF – SMF). Due to the large variation in their fat content, fish provide significantly different amounts of calories, and one serving covers 6.8–27.1% of daily energy requirement (SFF – SMF). Similar dietary values of fish meat were reported by Gokoglu et al. [22] and Erkan and Özden [23].

The smoking process increased the level of protein in one fish serving even up to 18–27% (FCS, SFS and FSAS, FTS, SBS, and SRS). Concurrently, there was a reduced amount of fat, i.e., 0.7–19.9% of daily fat requirement (SCS, FTS, and SMS). In agreement with these results, similar changes in the content of essential nutrients in thermally treated fish were reported by Bastías et al. [24].

The tissues of the fresh and smoked fish contained reasonable concentrations of sodium, potassium, calcium, magnesium, phosphorus, zinc, and copper. Although fish in this form are not perceived as a rich source of mineral elements, they should not be disregarded in an optimally balanced diet.

Fresh fish provide small amounts of Na, in comparison with the 0.5 g recommended as a minimum dose in a low-sodium diet, which is beneficial given the current salt-rich nutrition model [32]. However, the use of brine prior to fish smoking increased the sodium content over the recommended amount, on average even to 0.73 g in the freshwater fish (FSAS, FTS, and FWS).

In terms of K, one serving of fish covers approximately 23% and 12% of the recommended dietary amount of this element (freshwater and sea fish, respectively) [31]; this value, however, has been found insufficient for dietary cardiovascular prophylaxis [35].

Fish meat provides some amounts of Ca in the range of 7.5 to 5.0% and 12% of Mg of the daily requirement recommended by the Food Standards for the Polish Population [31]. The mean Mg content in fish covers approximately 17% of the level required to reduce the risk of hearing loss [36]. Raw or processed fish provide small amounts of P in the diet, covering only ~4% of the daily intake recommended in Polish diets and ca. 2% established for American diets [31,37].

The freshwater species analyzed in the study had a higher level of Zn (*p* < 0.05) than the sea fish (Table 3). Their meat contained on average 0.88 mg of the element per one serving, which corresponds to 4.7–8% of the daily requirement (Table 4). In turn, the sea fish were found to provide from 6.8 to 12.5% of the daily requirement of Zn recommended by European and Polish nutritional standards [2,31]. The fish species analyzed in the present study exhibited low amounts of Cu, i.e., on average 0.06 mg·kg^−1^, regardless of their origin, which on average covered 6.7% of the daily requirement [31] and only 2.5% of the level required for maintenance of good health status through lifetime [38]. Observations of the relationship between consumption, balance, and biomarkers of the Cu status performed in the French population since 1990 have indicated that the daily intake below 0.8 mg/day can lead to Cu losses and adverse health consequences throughout lifetime. The results of these analyses show that the Cu intake above 2.4 mg/day ensures long-term health benefits for consumers. However, the authors warn that high Cu concentrations are toxic, which impedes establishment of the dietary Cu requirement [38].

## 4. Conclusions

Both fresh and smoked fish are a good source of protein. The highest levels of protein were determined in bream and sea bream. Some species, like salmon, halibut, and mackerel are characterized by relatively high fat content. Bream, crucian, whitefish, flounder, herring, and red gurnard turned out to have the best nutritional value. The smoking process reduced the content of water, which contributed to the relative increase in the concentration of nutrients, including crude ash, and crude protein, and reduced the fat content. The content of mineral elements determined in the fish meat, i.e., K, Ca, Mg, P, Zn, and Cu, was reduced. The whitefish, trout, and sea fish—halibut, mackerel, and herring—were freshwater fish species with the highest levels of the analyzed minerals.

## Figures and Tables

**Table 1:** Scientific and common name of experimental fish and abbreviations in the experimental scheme.

Fish Name	Scientific Name	Polish	Source ^a^	Abbreviation in Experimental Scheme	
				Fresh	Smoked
Freshwater fish					
Bream	*Abramis brama*	Leszcz	Polish fish farm	FBF	FBS
Crucian	*Carassius carassius*	Karaś	Polish fish farm	FCF	FCS
Salmon	*Salmo salar*	Łosoś	Polish fish farm	FSAF	FSAS
Silver carp	*Hypophthalmichthys molitrix*	Tołpyga	Polish fish farm	FSIF	FSIS
Trout	*Oncorhynchus mykiss*	Pstrąg, Troć	Polish fish farm	FTF	FTS
Whitefish	*Coregonus lavaretus*	Sieja, Głąbiel, Brzona	Polish fish farm	FWF	FWS
Sea and ocean fish					
Sea bream	*Sparus aurata*	Dorada, Sparus Złotogłowy	Mediterranean sea	SBF	SBS
Cod	*Gadus morhua callarias*	Dorsz, Pomuchla	Baltic sea	SCF	SCS
Flounder	*Pleuronectesflesus*	Flądra, Płastuga, Stornia	Baltic sea	SFF	SFS
Halibut	*Hippoglossus hippoglossus*	Halibut, Kulbak	Atlantic Ocean	SHAF	SHAS
Herring	*Clupea harengus membras*	Śledź, Sałaka	Baltic sea	SHEF	SHES
Mackerel	*Scomber scombrus*	Makrela	Baltic sea	SMF	SMS
Red gurnard	*Chelidonichthys lucerna*	Kurek czerwony	Baltic sea	SRF	SRS

^a^ According to the information from the seller.

**Table 2:** Basic nutrients (g·kg^−1^ fish) and pH of fresh and smoked fish.

Fish	Dry matter	Crude ash	Crude protein ^a^	Ether extract ^b^	Energy ^c^ (kcal)	Energy ^c^ (kJ)	pH
Freshwater fish							
FBF	214.6 ± 0.53	11.9 ± 0.21	197.7 ± 0.17	10.5 ± 0.37	88.5 ± 0.32	371 ± 0.41	6.3 ± 0.47
FBS	274.3 ± 0.36	28.3 ± 0.33	226.1 ± 0.41	9.8 ± 0.14	99.3 ± 0.26	416 ± 0.27	6.1 ± 0.19
FCF	204.0 ± 0.54	11.6 ± 0.24	121.1 ± 0.25	24.5 ± 0.21	70.5 ± 0.26	295 ± 0.31	5.9 ± 0.31
FCS	245.6 ± 0.21	25.3 ± 0.28	163.9 ± 0.47	20.7 ± 0.14	84.2 ± 0.39	352 ± 0.34	5.7 ± 0.34
FSAF	353.7 ± 0.17	11.3 ± 0.23	189.2 ± 0.14	158.1 ± 0.32	218.0 ± 0.16	913 ± 0.24	6.5 ± 0.21
FSAS	242.8 ± 0.38	39.2 ± 0.15	258.9 ± 0.27	132.5 ± 0.25	222.8 ± 0.34	933 ± 0.19	6.4 ± 0.18
FSIF	190.3 ± 0.23	10.2 ± 0.43	168.3 ± 0.32	15.6 ± 0.29	81.4 ± 0.19	341 ± 0.18	6.3 ± 0.13
FSIS	241.3 ± 0.38	19.8 ± 0.41	187.2 ± 0.53	13.4 ± 0.13	86.9 ± 0.42	364 ± 0.15	6.1 ± 0.25
FTF	261.1 ± 0.12	18.3 ± 0.25	186.8 ± 0.13	7.9 ± 0.26	80.9 ± 0.18	339 ± 0.57	5.9 ± 0.16
FTS	325.3 ± 0.21	30.6 ± 0.19	241.7 ± 0.24	6.5 ± 0.31	103.5 ± 0.16	433 ± 0.24	5.7 ± 0.19
FWF	208.1 ± 0.18	11.1 ± 0.32	194.1 ± 0.26	12.2 ± 0.54	88.6 ± 0.25	371 ± 0.13	6.4 ± 0.34
FWS	279.4 ± 0.31	21.6 ± 0.28	223.4 ± 0.19	11.7 ± 0.18	99.9 ± 0.39	418 ± 0.17	6.3 ± 0.24
Sea and ocean fish							
SBF	282.6 ± 0.23	13.7 ± 0.19	216.9 ± 0.21	62.9 ± 0.16	143.4 ± 0.31	600 ± 0.27	6.6 ± 0.24
SBS	315.4 ± 0.34	23.6 ± 0.27	257.3 ± 0.38	51.3 ± 0.17	149.1 ± 0.45	624 ± 0.16	6.4 ± 0.31
SCF	192.8 ± 0.51	11.2 ± 0.31	184.9 ± 0.16	8.1± 0.31	81.3 ± 0.28	340 ± 0.34	6.3 ± 0.17
SCS	231.9 ± 0.19	16.8 ± 0.28	209.7 ± 0.45	6.4 ± 0.54	89.6 ± 0.39	375 ± 0.25	6.2 ± 0.24
SFF	159.2 ± 0.31	6.7 ± 0.16	145.2 ± 0.18	11.2 ± 0.16	68.2 ± 0.41	285 ± 0.18	6.4 ± 0.28
SFS	184.6 ± 0.17	9.3 ± 0.27	167.5 ± 0.24	9.7 ± 0.28	75.7 ± 0.56	317 ± 0.34	6.5 ± 0.54
SHAF	341.8 ± 0.24	12.7 ± 0.18	128.3 ± 0.37	163.2 ± 0.31	198.2 ± 0.48	830 ± 0.27	6.7 ± 0.19
SHAS	384.2 ± 0.38	18.4 ± 0.37	195.6 ± 0.16	153.2 ± 0.62	216.1 ± 0.19	905 ± 0.18	6.5 ± 0.33
SHEF	343.2 ± 0.16	11.5 ± 0.42	170.8 ± 0.31	102.9 ± 0.22	160.9 ± 0.31	674 ± 0.33	6.3 ± 0.18
SHES	381.7 ± 0.24	28.3 ± 0.51	233.6 ± 0.27	116.2 ± 0.17	198.0 ± 0.19	829 ± 0.47	6.1 ± 0.37
SMF	466.1 ± 0.18	9.2 ± 0.28	185.5 ± 0.18	198.5 ± 0.39	252.9 ± 0.28	1059 ± 0.38	6.3 ± 0.26
SMS	563.8 ± 0.23	14.6 ± 0.19	231.8 ± 0.46	141.1 ± 0.24	219.7 ± 0.34	920 ± 0.17	6.1 ± 0.19
SRF	200.5 ± 0.34	9.9 ± 0.34	183.3 ± 0.15	15.2 ± 0.37	87.0 ± 0.27	364 ± 0.28	6.5 ± 0.18
SRS	254.3 ± 0.16	17.3 ± 0.16	245.1 ± 0.27	10.4 ± 0.19	107.4 ± 0.16	450 ± 0.34	6.3 ± 0.22
ANOVA *p*-value ^c^	0.029	0.018	0.034	0.028	0.043	0.016	0.235
Global effect of smoking and the environment of fish occurrence							
MANOVA *p*-value	Raw/smoked fish	<0.001					
	Freshwater/sea and ocean fish	<0.024					
	Interaction effects	<0.015					

Results are the average ± standard deviation (*n* = 10 × 3 repetitions of chemical analyses). ^a^ Calculated by Kjeldhal nitrogen N × 6.25. ^b^ ether extract—crude fat determined with the Soxhlet method. ^c^ calculated for 100 g fresh matter of fish. ^c^ *p* < 0.05, statistical differences.

**Table 3:** Macroelements^a^ (g·kg^−1^ fish) and microelements^b^ (mg·kg^−1^ fish) of fresh and smoked fish.

Fish	Na ^a^	K ^a^	Ca ^a^	Mg ^a^	P ^a^	Zn ^b^	Cu ^b^
Freshwater fish							
FBF	0.43 ± 0.52	3.05 ± 0.26	0.62 ± 0.17	0.29 ± 0.37	2.14 ± 0.34	4.39 ± 0.71	0.41 ± 0.37
FBS	3.21 ± 0.26	2.79 ± 0.53	0.24 ± 0.51	0.15 ± 0.24	2.03 ± 0.46	3.78 ± 0.27	0.39 ± 0.11
FCF	0.35 ± 0.53	2.81 ± 0.24	1.03 ± 0.25	0.25 ± 0.27	2.16 ± 0.23	9.88 ± 0.31	0.43 ± 0.21
FCS	2.46 ± 0.21	2.36 ± 0.21	0.59 ± 0.44	0.19 ± 0.14	2.09 ± 0.39	6.73 ± 0.34	0.38 ± 0.34
FSAF	0.52 ± 0.17	3.78 ± 0.33	0.32 ± 0.14	1.09 ± 0.42	2.94 ± 0.26	5.30 ± 0.14	0.58 ± 0.41
FSAS	2.23 ± 0.38	2.12 ± 0.15	0.19 ± 0.57	0.54 ± 0.25	2.06 ± 0.34	4.32 ± 0.13	0.56 ± 0.18
FSIF	0.27 ± 0.21	2.92 ± 0.53	0.29 ± 0.37	0.23 ± 0.21	1.92 ± 0.11	4.28 ± 0.28	0.26 ± 0.19
FSIS	3.38 ± 0.32	2.31 ± 0.44	0.16 ± 0.13	0.21 ± 0.23	1.23 ± 0.32	3.97 ± 0.15	0.23 ± 0.25
FTF	0.43 ± 0.19	3.29 ± 0.25	0.21 ± 0.17	0.32 ± 0.27	2.26 ± 0.14	5.02 ± 0.37	0.31 ± 0.26
FTS	4.58 ± 0.31	2.76 ± 0.19	0.18 ± 0.34	0.17 ± 0.51	0.93 ± 0.16	4.12 ± 0.24	0.26 ± 0.14
FWF	0.43 ± 0.18	5.09 ± 0.12	0.73 ± 0.26	0.28 ± 0.34	2.87 ± 0.35	9.95 ± 0.13	0.53 ± 0.35
FWS	4.83 ± 0.31	4.23 ± 0.26	0.56 ± 0.19	0.21 ± 0.18	1.56 ± 0.49	8.73 ± 0.17	0.46 ± 0.24
Sea and ocean fish							
SBF	0.58 ± 0.23	4.12 ± 0.19	0.17 ± 0.21	0.34 ± 0.36	2.68 ± 0.31	4.83 ± 0.27	0.28 ± 0.26
SBS	3.46 ± 0.34	2.76 ± 0.25	0.06 ± 0.18	0.23 ± 0.17	2.09 ± 0.55	3.64 ± 0.16	0.26 ± 0.31
SCF	0.79 ± 0.41	2.97 ± 0.41	0.23 ± 0.12	0.27 ± 0.35	1.80 ± 0.38	3.99 ± 0.33	0.11 ± 0.27
SCS	1.89 ± 0.17	2.31 ± 0.28	0.17 ± 0.35	0.34 ± 0.54	1.16 ± 0.31	4.13 ± 0.25	0.09 ± 0.24
SFF	0.53 ± 0.31	1.40 ± 0.19	0.49 ± 0.18	0.16 ± 0.26	1.18 ± 0.21	4.26 ± 0.18	0.26 ± 0.28
SFS	2.59 ± 0.15	1.97 ± 0.37	0.37 ± 0.34	0.19 ± 0.21	1.21 ± 0.56	4.12 ± 0.14	0.31 ± 0.51
SHAF	0.75 ± 0.34	4.31 ± 0.15	0.09 ± 0.05	0.18 ± 0.31	2.56 ± 0.43	5.42 ± 0.23	0.41 ± 0.31
SHAS	2.09 ± 0.31	5.64 ± 0.27	0.12 ± 0.16	0.22 ± 0.42	2.64 ± 0.29	4.39 ± 0.48	0.38 ± 0.43
SHEF	1.06 ± 0.17	3.34 ± 0.43	0.61 ± 0.35	0.31 ± 0.29	2.49 ± 0.31	8.33 ± 0.43	0.92 ± 0.14
SHES	2.17 ± 0.24	4.56 ± 0.31	0.67 ± 0.27	0.27 ± 0.27	3.08 ± 0.15	6.25 ± 0.17	1.03 ± 0.27
SMF	1.12 ± 0.28	3.26 ± 0.23	0.15 ± 0.14	0.82 ± 0.34	2.36 ± 0.28	7.05 ± 0.34	0.81 ± 0.26
SMS	2.53 ± 0.43	4.15 ± 0.29	0.19 ± 0.36	0.61 ± 0.34	2.47 ± 0.44	6.69 ± 0.17	0.67 ± 0.39
SRF	0.78 ± 0.31	2.61 ± 0.37	0.33 ± 0.25	0.21 ± 0.37	1.62 ± 0.21	3.83 ± 0.18	0.07 ± 0.06
SRS	2.13 ± 0.16	2.89 ± 0.16	0.29 ± 0.27	0.17 ± 0.11	1.94 ± 0.36	3.25 ± 0.34	0.05 ± 0.04
ANOVA *p*-value ^c^	0.034	0.026	0.042	0.023	0.055	0.016	0.316
Global effect of smoking and the environment of fish occurrence							
MANOVA *p*-value	Raw/smoked fish	<0.001					
	Freshwater/sea and ocean fish	<0.016					
	Interaction effects	<0.008					

Results are the average ± standard deviation (*n* = 10 × 3 repetitions of chemical analyses). ^c^ *p* < 0.05, statistical differences.

**Table 4:** Calculated percent coverage of daily supply for selected nutrients and macro and microelements by consuming one serving ^a^ of fresh and smoked fish.

Fish	Protein	Fat	Energy	Na	K	Ca	Mg	P	Zn	Cu
Freshwater fish										
FBF	19.77	1.05	8.85	0.06	0.46	0.09	0.04	0.32	0.66	0.06
FBS	22.61	0.98	9.93	0.48	0.42	0.04	0.02	0.30	0.57	0.06
FCF	12.11	2.45	7.05	0.05	0.42	0.15	0.04	0.32	1,48	0.06
FCS	16.39	2.07	8.42	0.37	0.35	0.09	0.03	0.31	1,01	0.06
FSAF	18.92	15.81	21.8	0.08	0.57	0.05	0.16	0.44	0.80	0.09
FSAS	25.89	13.25	22.28	0.33	0.32	0.03	0.08	0.31	0.65	0.08
FSIF	16.83	1.56	8.14	0.04	0.44	0.04	0.03	0.29	0.64	0.04
FSIS	18.72	1.34	8.69	0.51	0.35	0.02	0.03	0.18	0.60	0.03
FTF	18.68	0.79	8.09	0.06	0.49	0.03	0.05	0.34	0.75	0.05
FTS	24.17	0.65	10.35	0.69	0.41	0.03	0.03	0.14	0.62	0.04
FWF	19.41	1.22	8.86	0.06	0.76	0.11	0.04	0.43	1.49	0.08
FWS	22.34	1.17	9.99	0.72	0.63	0.08	0.03	0.23	1.31	0.07
Sea and ocean fish										
SBF	21.69	6.29	14.34	0.09	0.62	0.03	0.05	0.40	0.72	0.09
SBS	25.73	5.13	14.91	0.52	0.41	0.01	0.03	0.31	0.55	0.52
SCF	18.49	0.81	8.13	0.12	0.45	0.03	0.04	0.27	0.60	0.12
SCS	20.97	0.64	8.96	0.28	0.35	0.03	0.05	0.17	0.62	0.28
SFF	14.52	1.12	6.82	0.08	0.21	0.07	0.02	0.18	0.64	0.08
SFS	16.75	0.97	7.57	0.39	0.30	0.06	0.03	0.18	0.62	0.39
SHAF	12.83	14.32	19.82	0.11	0.65	0.01	0.03	0.38	0.81	0.11
SHAS	19.56	18.32	21.61	0.31	0.85	0.02	0.03	0.40	0.66	0.31
SHEF	17.08	10.29	10.29	0.16	0.50	0.09	0.05	0.37	1.25	0.16
SHES	23.36	11.62	19.80	0.33	0.68	0.10	0.04	0.46	0.94	0.33
SMF	18.55	19.85	20.12	0.17	0.49	0.02	0.12	0.35	1.06	0.17
SMS	23.18	14.11	27.14	0.38	0.62	0.03	0.09	0.37	1.00	0.38
SRF	18.33	1.52	8.27	0.12	0.39	0.05	0.03	0.24	0.57	0.12
SRS	24.51	1.04	11.17	0.32	0.43	0.04	0.03	0.29	0.49	0.32
Daily intake	91 ^b^ g	65 ^b^ g	2000 ^b^ kcal	0.5 ^d^–3 ^e, f^ g	2 ^c^–2.9 ^g^ g	0.8 ^c^ g	0.3 ^h^–0.42 ^c^ g	0.7 ^c^–1.6 ^i^ g	6 ^j^–11 ^c,j^ mg	0.9 ^c^–2.4 ^k^ mg

^a^ One serving: 150 g, accepted as average fillet weight [3]. ^b^ Dietary Guidelines for Americans [3]. ^c^ Food Standards for the Polish Population [31]. ^d^ Minimal salt intake in low-sodium diets [32]. ^e^ Prevention of cardiovascular disease [33]. ^f^ The average daily sodium intake in the UK and northern European countries [34]. ^g^ The recommended amount in the diet of patients with cardiac diseases [35]. ^h^ Dietary Mg intake reducing risk of hearing loss [36]. ^i^ Estimated phosphorus intakes with diets in USA [37]. ^j^ EFSA; Scientific Opinion on Dietary Reference Values for zinc [2]. ^k^ The level at which good health status was observed in the 25 year studies [38].

## References

[1] Bainy E.M., Bertan L.C., Corazza M.L., Lenzi M.K. (2015). Effect of grilling and baking on physicochemical and textural properties of tilapia (*Oreochromis niloticus*) fish burger. J. Food Sci. Technol..

[2] European Food Safety Authority (EFSA) (2014). Scientific Opinion on Dietary Reference Values for zinc. EFSA.

[3] USDA, U.S. Department of Health and Human Services and U.S. Department of Agriculture (2015). 2015–2020 Dietary Guidelines for Americans. 8th Edition. https://health.gov/dietaryguidelines/2015/guidelines/.

[4] Wang D.H., Jackson J.R., Twining C., Rudstam L.G., Zollweg-Horan E., Kraft C., Brenna J.T. (2016). Saturated branched chain, normal odd-carbon-numbered, and n-3 (omega-3) polyunsaturated fatty acids in freshwater fish in the northeastern United States. J. Agric. Food Chem..

[5] Al-Yasiry A.R.M., Kiczorowska B., Samolińska W. (2017). The *Boswellia serrata* resin in broiler chicken diets and mineral elements content and meat nutritional value. Biol. Trace Elem. Res..

[6] Grela E.R., Kiczorowska B., Samolińska W., Kiczorowski P., Rybiński W., Hanczakowska E. (2017). Chemical composition of chosen leguminous. Part I. Basic nutrients, amino acids, antynutritional factors and antioxidant activity. Eur. Food Res. Technol..

[7] Emre N., Uysal K., Emre Y., Kavasoglu M., Aktaş Ö (2018). Seasonal and Sexual Variations of Total Protein, Fat and Fatty Acid Composition of an Endemic Freshwater Fish Species (Capoeta antalyensis). Aquat. Sci. Eng..

[8] Kiczorowska B., Kiczorowski P. (2011). Comparison of chemical composition and Mg, K, Na, Ca, Mn, Fe content in edible parts of chosen pear cultivars produced in podkarpackie province. Acta. Sci. Pol-Hortoru..

[9] Kiczorowska B., Klebaniuk R., Bąkowski M., Al-Yasiry A.R.M. (2015). Culinary herbs – nutritive value and content of minerals. J. Elem..

[10] Samolińska W., Kiczorowska B., Kwiecień M., Rusinek-Prystupa E. (2017). Determination of minerals in herbal infusions promoting weight loss. Biol. Trace Elem. Res..

[11] Mohanty B.P., Sankar T.V., Ganguly S., Mahanty A., Anandan R., Chakraborty K., Asha K.K. (2016). Micronutrient composition of 35 food fishes from India and their significance in human nutrition. Biol. Trace Elem. Res..

[12] García-Arias M.T., Alvarez-Pontes E., García-Linares M.C., García-Fernández M.C., Sánchez-Muniz F.J. (2003). Cooking–freezing–reheating (CFR) of sardine (*Sardina pilchardus*) fillets. Effect of different cooking and reheating procedures on the proximate and fatty acid compositions. Food Chem..

[13] Abraha B., Admassu H., Mahmud A., Tsighe N., Shui X.W., Fang Y. (2018). Effect of processing methods on nutritional and physicochemical composition of fish: A review. MOJ Food Process Technol..

[14] Cieślik I., Migdał W., Topolska K., Mickowska B., Cieślik E. (2018). Changes of amino acid and fatty acid profile in freshwater fish after smoking. J. Food Process Preserv..

[15] Famurewa J.A.V., Akise O.G., Ogunbodede T. (2017). Effect of storage methods on the nutritional qualities of African Catfish *Clarias gariepinus* (Burchell, 1822). Afr. J. Food Sci..

[16] Katola A., Kapute F. (2017). Nutrient composition of solar dried and traditionally smoked Oreochromis mossambicus (Peters, 1852). Int. Food Res. J..

[17] (2011). EU Commission Regulations No. 835/2011, 19.08.2011, amending the regulation (WE) No. 1881/2006 as regards the maximum levels for polycyclic aromatic hydrocarbons in foodstuffs. Dz. Urz. UE L 215/4–8. https://eur-lex.europa.eu/LexUriServ/LexUriServ.do?uri=OJ:L:2011:215:0004:0008:EN:PDF.

[18] Horwitz W., Latimer G.W., AOAC (2011). Official Methods of Analysis of AOAC International.

[19] Law Journal. UE L 304, 2011. Regulation (EU) No 1169/2011 of the European Parliament and of the Council of 25 October 2011 on the provision of food information to consumers, amending Regulations (EC) No 1924/2006 and (EC) No 1925/2006 of the European Parliament and of the Council and repealing Commission Directive 87/250/EEC, Council Directive 90/496/EEC, Commission Directive 1999/10/EC, Directive 2000/13/EC of the European Parliament and of the Council, Commission Directives 2002/67/EC and 2008/5/EC and Commission Regulation (EC) No. 608/2004, Annex XIV “Conversion Rates”. https://eur-lex.europa.eu/LexUriServ/LexUriServ.do?uri=OJ:L:2011:304:0018:0063:EN:PDF/.

[20] Manthey M., Karnop G., Rehbein H. (1988). Quality changes of European catfish (*Silurus glanis*) from warm water aquaculture during storage in ice. Int. J. Food Sci. Technol..

[21] PN-EN ISO 6869 (2002). Animal Feeding Stuffs—Determination of the Contents of Calcium, Copper, Iron, Magnesium, Manganese, Potassium, Sodium and Zinc—Method Using Atomic Absorption Spectrometry.

[22] Gokoglu N., Yerlikaya P., Cengiz E. (2004). Effects of cooking methods on the proximate composition and mineral contents of rainbow trout (*Oncorhynchus mykiss*). Food Chem..

[23] Erkan N., Özden Ö. (2007). Proximate composition and mineral contents in aqua cultured sea bass (*Dicentrarchus labrax*), sea bream (*Sparus aurata*) analyzed by ICP-MS. Food Chem..

[24] Bastías J.M., Balladares P., Acuña S., Quevedo R., Muñoz O. (2017). Determining the effect of different cooking methods on the nutritional composition of salmon (*Salmo salar*) and chilean jack mackerel (*Trachurus murphyi*) fillets. PLoS ONE.

[25] Marimuthu K., Thilaga M., Kathiresan S., Xavier R., Mas R.H.M.H. (2012). Effect of different cooking methods on proximate and mineral composition of striped snakehead fish (*Channa striatus, Bloch*). J. Food Sci. Technol..

[26] Bochi V.C., Weber J., Ribeiro C.P., Victório A.M., Emanuelli T. (2008). Fish burgers with silver catfish (*Rhamdia quelen*) filleting residue. Bioresource Technol..

[27] Vanitha M., Dhanapal K., Reddy G.V.S. (2013). Quality changes in fish burger from Catla (*Catla Catla*) during refrigerated storage. J. Food Sci. Technol..

[28] Fawole O.O., Ogundiran M.A., Ayandiran T.A., Olagunju O.F. (2007). Mineral composition in some selected fresh water fishes in Nigeria. J. Food Safety.

[29] Uran H., Gokoglu N. (2014). Effects of cooking methods and temperatures on nutritional and quality characteristics of anchovy (*Engraulis encrasicholus*). J. Food Sci. Technol..

[30] Ersoy B., Ozeren A. (2009). The effect of cooking methods on mineral and vitamin contents of African catfish. Food Chem..

[31] Jarosz M. (2012). Standards of Nutrition for the Polish Population—Revision (In Polish).

[32] He F.J., MacGregor G.A. (2009). A comprehensive review on salt and health and current experience of worldwide salt reduction programmes. J. Hum. Hypertens..

[33] WHO (2007). World Health. Prevention of cardiovascular disease: Guidelines for assessment and management of cardiovascular risk. https://www.who.int/cardiovascular_diseases/guidelines/Full%20text.pdf.

[34] Aburto N.J., Ziolkovska A., Hooper L., Elliott P., Cappuccio F.P., Meerpohl J.J. (2016). Effect of lower sodium intake on health: Systematic review and meta-analyses. Brit. Med. J..

[35] Ndanuko R.N., Tapsell L.C., Charlton K.E., Neale E.P., O’Donnell K.M., Batterham M.J. (2017). Relationship between sodium and potassium intake and blood pressure in a sample of overweight adults. Nutrition.

[36] Choi Y.H., Miller J.M., Tucker K.L., Hu H., Park S.K. (2014). Antioxidant vitamins and magnesium and the risk of hearing loss in the US general population. Am. J. Clin. Nutr..

[37] Calvo M.S., Moshfegh A.J., Tucker K.L. (2014). Assessing the health impact of phosphorus in the food supply: Issues Organization and considerations. Adv. Nutr..

[38] Bost M., Houdart S., Oberli M., Kalonji E., Huneau J.F., Margaritis I. (2016). Dietary copper and human health: Current evidence and unresolved issues. J. Trace Elem. Med. Bio..

